# Nipple stimulation for labour augmentation: evidence from randomised and quasi-experimental studies

**DOI:** 10.1186/s12884-025-08393-3

**Published:** 2025-12-01

**Authors:** Matilda Videgård, Louise Anderberg, Michael B. Wells

**Affiliations:** Department of Women’s and Children’s Health, Karolinska Institutet, Floor 8, Elevator 1, Tomtebodavägen 18A, Stockholm, 171 77 Sweden

**Keywords:** Nipple stimulation, Breast stimulation, Birth, Labour augmentation, Literature review

## Abstract

**Background:**

Physiological childbirth is associated with a safer birth, including reducing unnecessary medical interventions, and having a more positive birthing experience. Synthetic oxytocin, commonly used for labour augmentation, carries risks such as neonatal complications, uterine hyperstimulation, and postpartum haemorrhage. Nipple stimulation (NS) may offer a safe, non-pharmaceutical alternative for labour augmentation.

**Aim:**

To explore the effectiveness and safety of nipple stimulation used for labour augmentation in women with spontaneous contractions or rupture of the membranes.

**Method:**

A literature review was conducted using PubMed, CINAHL, Web of Science, PsycInfo, Google Scholar, and Consensus.app. A narrative synthesis was conducted following the ESRC guidance for synthesizing quantitative evidence, using thematic analysis and structured exploration of relationships across studies. Calculations of Odds Ratios and Confidence Intervals were made where permissible.

**Results:**

Ten studies (four RCTs, five quasi-experimental, one cross-sectional) involving 1251 women were included. NS was effective for augmenting labour in most studies, reducing synthetic oxytocin usage, increasing contraction frequency and Bishop scores, shortening the duration of labour stage one, and increasing the likelihood of spontaneous vaginal birth. NS appeared safe in the included studies, with few and reversible side effects reported. However, substantial heterogeneity in study designs, interventions, and measured outcomes limited comparability and precluded meta-analysis.

**Conclusions:**

Nipple stimulation appears to be safe and effective for labour augmentation. However, substantial heterogeneity in study designs and outcomes limits generalisability. Further large-scale randomised controlled trials are needed to establish clinical protocols and determine which populations benefit most.

**Supplementary Information:**

The online version contains supplementary material available at 10.1186/s12884-025-08393-3.

## Introduction

Avoiding unnecessary medical interventions during birth is linked to safer and more positive experiences for women [1–3]. However, synthetic oxytocin, a drug commonly used to augment labour, is used with 30–90% of birthing women globally, even though only 10–15% of births become prolonged in a way that poses an actual medical problem [4]. Additionally, there is no medical consensus and no international guidelines of when and in what dose to administer synthetic oxytocin [5]. Synthetic oxytocin poses health risks for both the mother and baby, with increased risks of stillbirth [4], uterine hyperstimulation [4, 6], neonatal complications [4, 7], bladder damage [7], postpartum haemorrhage [7, 8], and increasing the risk of having a negative birthing experience [7]. There is a movement among women and midwives to reduce interventions and promote physiological birth [9]. Given the growing interest in physiological approaches to birth, this review explores whether nipple stimulation (NS) could serve as a safe and effective method for labour augmentation. We hypothesise that NS may be a safe and effective method for labour augmentation that may reduce the need for synthetic oxytocin.

### The effects of nipple stimulation in the peripartum period

Labour augmentation is defined as any method used to stimulate contractions after the onset of labour to effect a vaginal delivery [10]. However, the onset of labour is not clearly defined [11]. For the purpose of this review, we operationally defined the onset of labour as either spontaneous rupture of membranes or the presence of spontaneously occurring contractions. One non-pharmaceutical way to stimulate contractions is through NS. NS refers to stimulating the nipples by contact, most commonly either manually with fingers or by applying a breast pump [12]. Two hours of NS can trigger contraction frequency similar to synthetic oxytocin, but with lower plasma oxytocin levels [13]. While NS contraction-stimulating effect has been known since Hippocrates [14], currently, NS is used for labour augmentation by Canadian midwives [15] and is recommended as a natural induction method in Japanese midwifery guidelines [16].

NS increases the release of endogenous oxytocin [13]. Endogenous oxytocin is one of the most important hormones for effective labour contractions [17], but it is not solely responsible for increasing contractions, as NS seems to trigger the complex hormonal response of physiological labour in a way that synthetic oxytocin does not [13]. While synthetic oxytocin acts only in the bloodstream, endogenous oxytocin is released both into the circulation and directly within the brain during labour [18]. Endogenous oxytocin released into the brain has several health benefits including decreasing pain, fear, and stress, increasing the release of endogenous opiates, as well as increasing the pregnant woman’s capacity to feel relaxed during birth and promoting mother-infant bonding post-birth [18]. These positive health effects are not seen with synthetic oxytocin, as synthetic oxytocin does not cross the blood-brain barrier [18].

NS has also shown to ripen the cervix [19–21], induce labour and decrease the number of women not in labour after 72 hours [12], and reduce postpartum haemorrhage [12, 22, 23]. While NS is useful for causing and improving uterine contractions [23–26], there appears to be no previous literature review on nipple stimulation for the purpose of labour augmentation. This review evaluates whether NS is a viable method for augmenting labour in women with spontaneous contractions or rupture of membranes. Since NS has previously been shown to promote cervical ripening, initiate labour, and reduce postpartum haemorrhage, we hypothesise that it may also support labour progression once labour has begun. Based on this, we hypothesise that NS could be effective for labour augmentation; thus, reducing the need for synthetic oxytocin, and is safe for both mother and baby.

## Method

### Study design

A literature review and narrative synthesis was conducted on the effects of NS used for labour augmentation. This review used a narrative synthesis approach, guided by the ESRC framework [27], which includes four key elements: (1) developing a theory of how the intervention works, (2) developing a preliminary synthesis, (3) exploring relationships in the data, and (4) assessing the robustness of the synthesis. This approach was chosen because heterogeneity in study designs and outcomes precluded meta-analysis.

### Search strategy

Two authors searched broadly for articles on using NS in the peripartum period to learn what had been published in the field. This helped the authors form the basis of their research question and selection criteria. Initial searches were conducted in PubMed, Cinahl, and consensus.app, an AI tool. From the initial broad searches, key articles were identified and studied, providing important insight on which inclusion and exclusion criteria would be relevant to answer our aim [27]. Any article, regardless of study design, that focused on NS for the purpose of labour augmentation, written in English, and published between 1960 and February 2024, were included. Studies using an intervention and control group that did not specify the obstetric status of those participants within those groups prior to the intervention were excluded, as the effects of the intervention could not be accurately measured.

The key articles also helped identify relevant search terms. We explored different combinations of search terms to both expand and narrow the search. Librarians employed at Karolinska Institutet were consulted on several occasions and guided the authors throughout the entire search process. Searches were conducted in PubMed, Cinahl, PsycInfo and Web of Science. A combination of thesaurus terms and free-text terms were used to maximise the number of relevant articles. Since thesaurus terms are specific for each database, terms relevant for each database were identified with the help of librarians. The search used two clusters of keywords:

1. The intervention, i.e. nipple stimulation.
2. The process studied, i.e. birth.

The two clusters were combined with the Boolean term AND, whereas thesaurus terms and free-text terms were combined within each cluster with OR. In PubMed, using only two keyword clusters made the search too broad, which was managed by using a third cluster of keywords added with NOT. The free-text terms included in the NOT-cluster were decided upon after reviewing the search hits and carefully selecting words that would not exclude any relevant articles. All free-text terms in the NOT-cluster were only excluded if found at the title level, except the word “cancer” which was excluded from both title and abstract. All searches and search trials are found in Supplementary File 1 and 2.

**Fig. 1:**
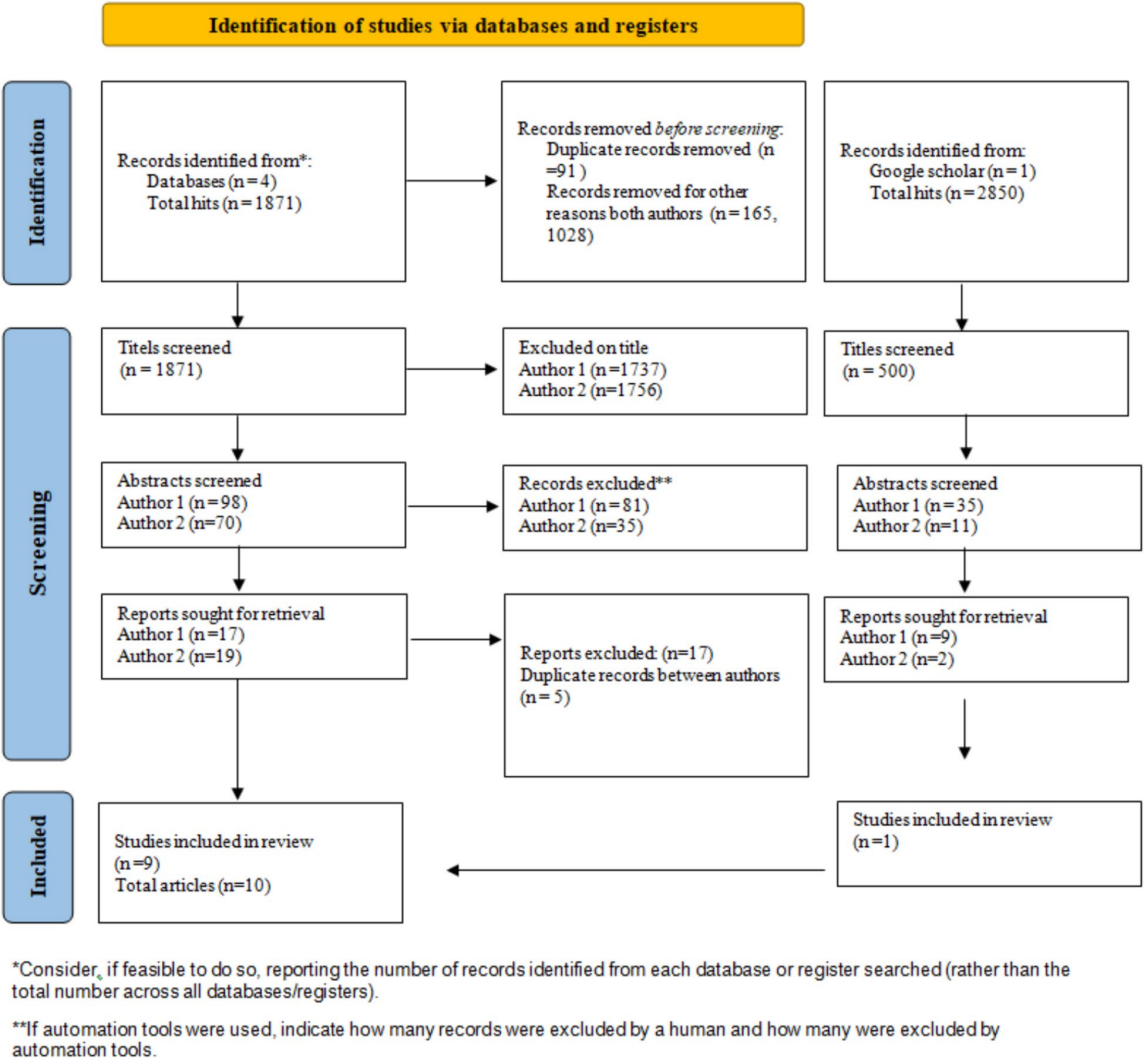
PRISMA (2020) flow chart

An additional search took place in Google Scholar, using a narrower search to limit the number of hits (Supplementary File 1). Since Google Scholar sorts the hits by relevance, only the first 500 hits were examined. Finally, consensus.app, a tool using artificial intelligence (AI), was searched. The authors have deliberately incorporated this tool into their free search methodology (Supplementary File 1) to ensure that relevant articles not covered by other databases were included [28]. The hits of the final search of each database were transferred to EndNote, where duplicates were removed. The final search is presented in a PRISMA flow chart, see Fig. 1. No new articles meeting the inclusion criteria were added from the Consensus.app search and therefore, Consensus.app is not represented in the PRISMA flowchart.

### Critical appraisal of included studies

All included studies were assessed individually by the first two authors and then discussed. Randomised controlled trials (RCTs) were assessed using the Critical Appraisal Skills Programme (CASP) [29]. The quasi-experimental studies were assessed using the JBI Checklist for Quasi Experimental Studies [30]. The one cross-sectional study was assessed using the STROBE checklist for cross-sectional studies [31]. While STROBE is primarily a reporting guideline, it was used here as a proxy to assess reporting completeness and methodological transparency. Discrepancies between reviewers were resolved through discussion with the third author. The number of studies that either totally met, partially met or not met each question asked in the appraisal tools were shown in Supplementary File 3. Based on the results of the critical appraisals, the included studies were generally of moderate to high methodological quality, with the exception of one study that was appraised as having lower quality.

### Data extraction

Data extracted from each article included location, design, aim, sample size, intervention details, obstetric status, parity, and ethics (Supplementary File 4).

### Data analysis

Two authors independently ran the searches, reviewed the articles and then read and discussed all potentially relevant articles in full text. To ensure no articles were missed, the reference list of each relevant article was scanned for additional material. When the authors were uncertain of whether to include an article or not, the third author, who is experienced in literature reviews, was consulted. All authors agreed on the included articles.

This review followed the ESRC guidance on narrative synthesis, which outlines four key elements: (1) developing a theory of how the intervention works, (2) developing a preliminary synthesis, (3) exploring relationships in the data, and (4) assessing the robustness of the synthesis. A theory of change was developed based on existing literature, positing that nipple stimulation promotes labour progression through the release of endogenous oxytocin and associated hormonal cascades. This theory informed the interpretation of findings and helped identify relevant outcomes.

The preliminary synthesis was conducted using thematic analysis by Braun and Clarke [32], which consists of six steps: 1) each study was read through several times to get a deeper understanding of the data, 2) initial codes of data relevant to the study’s aim were created, 3) initial codes were sorted into themes, 4) themes were reviewed, synthesised and discussed between authors, 5) themes were refined and organised into main themes and sub-themes, 6) main themes and sub-themes were presented in the Result section. Steps one to three were completed independently by two authors, while steps four to six were conducted in collaboration between all authors.

To explore relationships in the data, the authors examined how variations in study design, intervention type, and participant characteristics influenced outcomes. Studies were grouped and compared based on intervention method (e.g., manual stimulation vs. breast pump), duration, and obstetric status at baseline. This allowed for identification of patterns.

During the analysis, odds ratios (OR) were searched to include in the results. In the articles where the OR was not stated, then the OR was calculated, if data allowed. To ensure reliability, two authors checked the numbers of participants, and the calculation was performed in the mathematical program MedCalc [33].

### Ethical considerations

Ethical approval was not required as this review used only secondary data.

## Results

The searches generated 1871 articles from five databases, and one additional article was found through consensus.app. However, this article was written in a non-English language and so was excluded from further analysis. Ten articles, totalling 1251 women, met the inclusion criteria. As shown in Supplementary File 4, four were RCTs, five were quasi-experimental and one was a cross-sectional study. Two studies compared women practising NS with women receiving oxytocin, two had three groups comparing NS with uterine stimulation and a no-intervention group, two compared NS with those receiving no intervention, and four did not have control groups. Studies came from the USA (4), Israel (2), and one each from India, Iran, Egypt, and Turkey. Six articles were published before the year 2000, while the remaining four were published between 2015 and February 2024.

Three methods for NS were identified in the studies. The most common form of NS was manual stimulation, which included rolling, massaging, tweaking or pinching with hand or fingers, performed by the woman, the midwife, or a support person [34*, 35*, 36*, 37*, 38*]. Other articles reported using a breast pump either unilaterally or bilaterally [39*, 40*, 41*, 42*] and one article reported using electro stimulation with electropads on the areola area [43*].

**Fig. 2:**
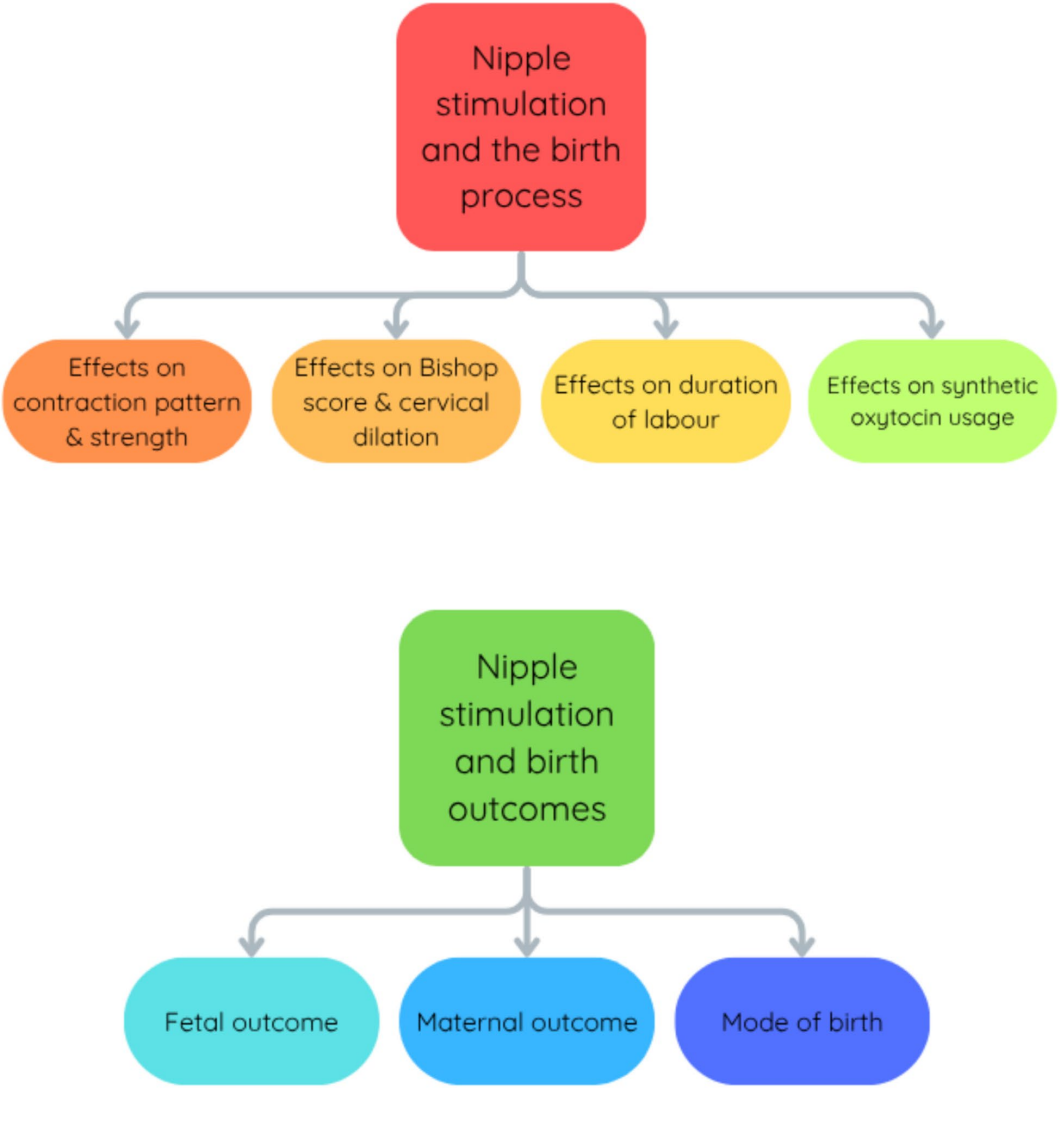
Showing the themes and sub-themes

The thematic analysis resulted in two main themes: *Nipple Stimulation and the Birth Process* and *Nipple stimulation and Birth Outcomes* (Fig. 2).

### Nipple stimulation and the birth process

All studies concluded that NS had augmenting effects on the birthing process for a large portion of women [34*, 35*, 36*, 37*, 38*, 39*, 40*, 41*, 42*, 43*]. The extent of the augmenting effects will be presented in the sub-themes below.

At the start of intervention, women either had a mean cervical dilation of 1–5 centimetres with some contractions [35*, 36*, 37*, 38*, 41*] or ruptured membranes with absent or infrequent contractions [35*, 37*, 39*, 40*, 42*, 43*].

#### Effects on contraction pattern and strength

All studies reported some augmenting effects on contraction patterns. An intrauterine pressure catheter was used in two studies [35*, 41*] and measured contraction intensity in montevideo units. Other methods of measuring effects on contraction patterns included clocking the duration and/or interval of contractions [35*, 36*, 37*, 38*, 39*, 41*, 42*]. Contraction intensity was also described in subjective terms such as “moderate” or “strong” [36*].

The effectiveness was different depending on if the study design allowed women to stimulate throughout the birth or for a limited amount of time (Supplementary File 4). Women with ruptured membranes who stimulated their nipples throughout the birth process were able to increase their contraction frequency with NS in 69–100% of cases [40*, 42*, 43*]. Of those studies that imposed a time restriction to NS, all fell between 30–60 minutes and the women in those studies either increased contraction frequency or their cervical dilation in 35–50% of cases [39*, 41*].

Contractions increased significantly in duration and interval in the intervention group compared with control groups receiving no intervention [36*, 38*]. The time it took to increase contraction frequency with NS ranged from 20–180 minutes, with a median of 100 minutes [40*, 42*, 43*] while the time with synthetic oxytocin was not reported. The median strength of contractions induced by NS was 48 mmHg to 68 montevideo units [35*, 41*], which was lower than the average strength of 101 montevideo units induced by synthetic oxytocin [41*]. For women with infrequent contraction patterns, NS was reported to increase contraction frequency, but not contraction strength [35*, 41*].

#### Effects on Bishop score and cervical dilation

Four studies reported on changes in Bishop scores and cervical dilation, and all of them found significant changes in the NS group compared with the control group [34*, 36*, 38*, 42*]. Median Bishop scores at 4–6 hours after intervention were 12.65–13 for the NS group and 10.82–11.5 for the control group receiving no intervention [34*, 36*]. Other reports of cervical changes included measuring dilation four hours after intervention, finding 83% of women in the NS group had 6–8 cm dilation, while 100% of women in the control group had 2–4 cm dilation [38*]. One study found that the difference in the Bishop scores had levelled out between the NS group and the control group by the sixth hour [36*].

#### Effects on duration of labour

Nipple stimulation shortened the duration of the first stage of labour compared with control groups receiving no intervention [34*, 36*, 37*, 38*, 43*]. The first stage of labour was significantly less for women in the NS group compared to the control group (3.8–5.5 hours [NS group], 6.8–7.3 hours [control group]) [34*, 37*]. Within 10 hours after the start of intervention, women in the NS group were more likely to have given birth compared to women in the control group (OR 26; CI 6.5–104.5) [38*] (OR 26; CI 1.5–460.5) [36*]. In studies that compared women practising NS with women receiving synthetic oxytocin infusion, no difference was found in total labour time [39*, 41*]. However, one study found that the time from intervention to birth was faster for women receiving synthetic oxytocin compared with women practising NS [39*].

A meta-analysis on NS’s effect on labour duration was not conducted due to the substantial heterogeneity in study design, population characteristics, and outcome reporting (see Table 1).

**Table 1:** Sample size, obstetric starting point, and labour duration

Article	Sample size NS vs CG or OI (Oxytocin Infusion)	Obstetric starting point	Labour duration	Statistical significance	Notes
Demirel [34*]	NS 130 vs CG 130	Avg. Bishop score 9/13; mixed parity	1 st stage avg. NS 3.8 +/- 39.9 vs CG 6.8 +/−118.9 2nd stage avg. NS 16.3 +/- 10.8 vs CG 27.3 +/−16.1	1 st stage *p* = 0.001 2nd stage *p* = 0.001	Article does not specify if time was measured from start of labour or start of intervention; start of intervention is assumed
Ibrahim [36*]	NS 46 vs CG 40	Avg. Bishop score 8/13; primiparas only	1 st stage avg. NS 4.4 ± 0.83 vs CG 6.3 ± 1.17 hrs 2nd stage avg. NS 18.7 +/- 4.1 min vs CG 24.6 +/- 5.0 min	1 st stage *p* >0.001 2nd stage *p* >0.001	Article does not specify if time was measured from start of labour or start of intervention; start of intervention is assumed
Mousavi [37*]	NS 110 vs CG 110	Avg. dilation 2.5 cm, minimum 2 contractions per 10 min.	Latent phase <6cm NS 3.2 (1.3–6.3.3.3) hrs vs CG 4.8 (0.8–3.0.8.0) hrs Active phase >6 cm NS 2.3 (1.4–3.0.4.0) hrs vs CG 2.5 (2.0–3.3.0.3) hrs.	Latent phase *p* = 0.008 Active phase *p* = 0.25	Article does not specify if time was measured from start of labour or start of intervention; start of intervention is assumed
Suja [38*]	NS 30 vs CG 30	2 cm dilation with mild contractions	1 st stage avg. NS <8 hrs: 20 8–10 hrs: 10 >10:0 vs CG <8 hrs: 0 8–10 hrs: 6 >10: 24	Not reported.	
Curtis [39*]	NS 48, OI 30	ROM 1–24 hrs, <5 cm dilation <1, contraction/5 min	Intervention to delivery NS 10.5 ± 4.2 hrs vs OI 6.6 ± 3.4 hrs	Intervention to delivery for primiparas *p* = 0.002 Intervention to delivery for multiparas *p* = 0.03	
Stein [41*]	NS 17, OI 48	NS dilation 5 +/- SD 2 cm OI dilation 5 +/- SD 3 cm	Intervention to delivery NS 5 +/- SD 2 hrs vs OI 6 +/- SD 4 hrs 1 st stage NS 15 +/- SD 6 hrs vs OI 14 +/- SD 6 hrs 2nd stage NS 46 +/- SD 50 min vs OI 42 +/- SD 50 min	Intervention to delivery *p* = 0.3 1 st stage *p* = 0.6 2nd stage *p* = 0.8	

#### Effects on synthetic oxytocin usage

Nipple stimulation was associated with a lower proportion of women who received synthetic oxytocin infusion compared to control groups (OR 1.7; CI 0.9–3.9) [37*] (OR 36; CI 11–122) [36*]. The rate of women subjected to synthetic oxytocin infusion ranged from 4.6–26.6% in the NS group to 38–92.3.3% in the control group receiving no intervention [34*, 36*, 37*].

### Nipple stimulation and birth outcomes

#### Fetal outcome

Half of the studies assessed fetal outcomes. There was no difference in Apgar scores between NS groups, control groups, or compared with women receiving synthetic oxytocin [37*, 39*, 41*, 43*]. The NS group had a lower number of children passing meconium during birth compared to those receiving synthetic oxytocin (RR; 0.2, CI; 0.04–1.3.04.3) [39*].

The only negative outcome concerned one participant in one study, who had an episode of fetal heart rate deceleration and tachycardia after practising NS [35*]. The stimulation was promptly stopped, leading to heart rate normalisation.

#### Maternal outcome

No adverse maternal outcomes were reported in any of the studies. Cases of uterine tachysystole and prolonged contractions, which were resolved within a few minutes after stopping NS, were mentioned in two studies [35*, 43*]. Uterine tachysystole was defined as more than five contractions per minute, and prolonged contractions were defined as lasting more than 90 seconds [35*, 43*]. Other studies reported no cases of uterine hyperstimulation [37*, 41*].

Breast-related side effects were mentioned in three studies. The reports included nipple soreness [42*] and that using NS during labour had a positive impact on subsequent breastfeeding [40*, 43*].

Maternal satisfaction was mentioned in two studies [42*, 43*], stating that women preferred NS because it gave them more control over labour [42*], and that they would be willing to experience NS again in subsequent labours [43*].

#### Mode of birth

Practising NS was generally associated with a higher proportion of spontaneous vaginal births compared to control groups. The OR of having a spontaneous vaginal birth with NS compared with no intervention was calculated in four studies and is presented in Table 2. Of these, two studies reported a statistically significant result [34*, 36*]. The other two studies showed trends in favor of NS but did not reach statistical significance [37*, 38*].

**Table 2:** OR for vaginal birth rates in NS groups versus control groups receiving no intervention

**Study**	**NS Vaginal Births (n)**	**NS Group** **(n)**	**Control Vaginal Births (n)**	**Control Group** **(n)**	**OR**	**95% CI**
Demirel & Guler [34*]	130	130	119	130	25	1.5–430.5
Ibrahim et al. [36*]	46	46	40	50	24.1	1.4–424.4
Suja [38*]	30	30	30	30	1	0.02–52.02
Mousavi et al. [37*]	47	110	42	110	1.2	0.7–2.07.7.07
Composite	253	316	231	320	1.6	1.07–2.24.07.24

To provide a more robust estimate, a composite analysis was calculated using pooled data from the four studies. Among 320 women in the NS group, 253 (79.1%) achieved spontaneous vaginal births, compared to 231 out of 320 women (72.2%) in the control groups. The overall odds ratio for spontaneous vaginal birth with NS was 1.45 (95% CI: 1.01–2.09), indicating a statistically significant benefit. This pooled analysis strengthens the evidence that NS may increase the likelihood of having a spontaneous vaginal birth and supports its consideration as a viable, non-pharmacological method for labour augmentation.

## Discussion

This literature review and narrative synthesis utilising ten quantitative studies on labour augmentation indicates that NS reduces the need for synthetic oxytocin, increases the chance of having a spontaneous vaginal birth, enhances contraction frequency, increases cervical dilation and Bishop scores, and shortens the first stage of labour. NS was most effective when practiced for over an hour. However, these results need to be interpreted with caution and more large-scaled randomised controlled trials are needed to determine in which way NS can be recommended in clinical settings. The varying study designs and the relatively small number of articles available for inclusion presented a challenge when conducting the review. There was a large variation of the technique, duration and interval of NS, and almost all studies measured different outcomes and had different obstetric starting points when beginning NS.

### Four uses of nipple stimulation

According to previous randomised controlled trials [19–21] and literature reviews [12, 22, 23], as well as this current review, NS may benefit birthing women in four main areas: 1) increasing cervical ripening before labour [19–21], 2) inducing labour with a favourable cervix [12], 3) potentially reducing postpartum haemorrhage, though evidence is conflicting [12, 22, 23], and 4) be an effective tool for labour augmentation.

To our knowledge, this is the first literature review to provide a broad and inclusive overview on NS’ impact on labour augmentation. NS is a non-pharmaceutical method birthing women can use and control themselves, while promoting a physiological birth. Therefore, NS may be applicable in home births, low‑resource settings, and hospitals when appropriate monitoring and protocols are in place. Additionally, NS avoids drug-related adverse effects associated with exogenous oxytocin; however, data on rare harms from NS remain limited [4, 6–8] and it may have a stronger effect on uterine contractility than other non-pharmaceutical methods focused on increasing the release of endogenous oxytocin [24, 44]. Using NS could decrease the number of women with labour dystocia, also known as failure to progress in labour, a common situation that increases the risk of having multiple interventions, such as transferring to hospital during a planned home birth [45], receiving a synthetic oxytocin infusion, and having an instrumental or caesarean birth [46].

### An alternative to synthetic oxytocin?

Recent studies emphasise the importance of education and exploring alternative approaches to reduce synthetic oxytocin usage without medical indication [47]. Educating healthcare providers about other techniques has significantly decreased oxytocin administration from 77.8% to 32.1% in India, showing that changing routines for synthetic oxytocin prescription is possible [47]. Similarly, one study reported that 78% of health professionals would prefer NS over synthetic oxytocin if studies conclude NS to be effective [39*].

A recent meta-analysis compared labour time for women practising NS vs women receiving synthetic oxytocin and found that labour length was 13.42 vs 12.46 hours, thus concluding that NS could be a comparable option for labour induction and augmentation [48]. The study was limited by the fact that a proportion of women in the NS group also received synthetic oxytocin, so conclusions regarding NS as a standalone alternative could not be drawn [48]. In our study, NS was effective for a majority of women regarding labour augmentation, however not all women benefited from NS and some were therefore given synthetic oxytocin. In our review, eight out of ten studies conclude that NS was effective for labour augmentation[34*, 35*, 36*,^37^*, 38*, 40*, 42*, 43*], and two other studies consider it effective, but only for 35–50% of participants [39*, 41*]. A recent RCT compared NS vs oxytocin infusion for women with a previous caesarean, and found that although the time from augmentation to delivery was longer for the NS group, the outcomes and effectiveness of the methods was otherwise comparable, with NS having significantly less cases of uterine tachysystole [49]. Synthetic oxytocin is commonly considered effective for labour augmentation, where research from the 1950 s suggests a success rate of 97.3% when used for labour augmentation [50], leading more modern research to focus on which regimen and dosage of oxytocin is most effective [5, 46]. According to the current review, NS could not replace synthetic oxytocin in all situations where labour augmentation is needed. However, since it is a non-pharmaceutical, safe, and effective method, which can be controlled by the woman herself in any setting to promote the hormonal response of physiological labour, including NS in the repertoire of labour augmentation methods could be beneficial for some birthing women. Before this can be recommended in clinical settings, future research needs to determine in which obstetric situations NS is likely to be effective and which protocol for NS is most useful, to formulate guidelines for its usage. Although existing RCTs and quasi-experimental studies indicate that NS can be effective and safe, the overall evidence base is limited in size and consistency due to heterogeneous protocols and outcomes. Consequently, routine, universal implementation is premature, and further high-quality, standardized trials are needed to confirm effectiveness, safety, and optimal protocols.

### Is NS safe?

One critical aspect to address in labour augmentation involving NS is the rare occurrence of hyperstimulation. A study from 1986 tested the ability to hyperstimulate with NS, and concluded that 2–21% were able to hyperstimulate [51]. In a case report, one woman presented at a clinic with uterine tachysystole and prolonged fetal heart rate decelerations after practising NS at home [52]. In other studies with NS, no hyperstimulation was found, and the method was seen as cost-effective and safe [26]. In our review, 1 in 1251 women experienced fetal heart rate decelerations due to uterine tachysystole when performing NS, which was then reversed by simply stopping NS. The risk of hyperstimulation is of increased importance for groups with increased risk for uterine rupture, such as grand multiparous women and women with a previous caesarean delivery, who are contraindicated for synthetic oxytocin infusion [53]. NS has been tested as a labour induction method for women with the above mentioned risk factors and was deemed safe and effective, with 84% achieving a vaginal birth [53]. Women with a previous caesarean who practised NS had no cases of uterine rupture, compared to two cases among women who received a synthetic oxytocin infusion, a recent RCT shows [49]. The current review suggests that NS is generally safe for mother and baby, but that hyperstimulation is possible in rare instances. Because of rare instances of fetal distress caused by hyperstimulation of the uterus after NS, any woman considering practising NS for labour augmentation purposes would be advised to consult with a midwife beforehand and to take proper safety precautions.

### Pain relief and women’s perspectives of NS

Two important areas were largely not reported on: 1) no studies reported on the use of pain relief in combination with NS and the possible interactions between the two, and 2) only two studies reported briefly on women’s views and experiences with NS [42*, 43*]. Further knowledge differentiating the effects of NS with and without analgesia, and women’s experiences of NS might impact in what situations and for which women NS is either encouraged or discouraged.

Three commonly used medical methods of pain relief in labour are inhaled nitrous oxide, opioids, and epidural analgesia. While inhaled nitrous oxide [54] and opioids [55] have their own set of pros and cons, they have not been reported to affect uterine contractility. Epidural analgesia, on the other hand, increases the length of the first and second stages of labour, as well as increasing the risk of receiving synthetic oxytocin, according to a Cochrane review including 40 studies and 11,000 women [56]. The four randomised controlled trials included in the current review were conducted in Iran, Turkey and the United States. Studies indicate that the percentage of women receiving epidural analgesia during labour at the time of the studies were around 37% in Iran [57], 57–96% in private clinics in Turkey [58], and 50% in the United States [59]. Unless any restrictions were placed on pain relief, and no studies reported having these restrictions, it is reasonable to think that a portion of participants had epidural analgesia. The fact that none of the studies investigated the difference of NS on a uterus affected by an external substance, like an epidural, with the effects of NS on a uterus labouring physiologically is an important missing piece in understanding the effects of NS regarding labour augmentation.

To our knowledge, in the 50+ years that NS has been studied for labour augmentation, we found no qualitative studies exploring women’s experiences of NS. Cursory searches also imply that no research has asked this question in relation to the three other uses of NS: improving cervical ripening, labour induction and preventing postpartum haemorrhage, either. Thus further research is warranted regarding women’s experiences of NS within this arena.

## Strengths and limitations

While the methodology of this literature review and narrative synthesis on nipple stimulation for labour augmentation is robust, it is not without limitations. Inherent subjectivity is always potentially problematic in qualitative analyses. This was mitigated via using a systematic approach and using thematic analysis completed by three researchers.

A major limitation was the considerable heterogeneity across the included studies. Study designs ranged from randomised controlled trials to quasi-experimental and cross-sectional studies. Intervention protocols varied widely in terms of stimulation method (manual, breast pump, electrostimulation), duration, and frequency. Baseline obstetric conditions (e.g., cervical dilation, membrane status) and outcome measures (e.g., contraction frequency, Bishop score, labour duration, mode of birth) also differed considerably. This variability precluded meta-analysis and complicated direct comparison of effect sizes, limiting the generalisability of findings. Future research should prioritise standardised protocols and outcome reporting to enable more robust synthesis.

Excluding non-English articles meant potentially losing important data affecting the results [60]. However, articles in all languages were included at the search level and some of them had abstracts in English. Most non-English abstracts indicated that NS was effective for labour augmentation [61–63], while one stated NS was not effective [64].

The exclusion of studies that did not specify obstetric status prior to intervention may have led to the omission of potentially relevant data; however, this decision was made to preserve the integrity and interpretability of the results. To evaluate the methodological rigor of the included studies, we used established critical appraisal tools appropriate to each study design: CASP for randomized controlled trials, the JBI Checklist for quasi-experimental studies, and the STROBE checklist for the cross-sectional study. While STROBE is primarily a reporting guideline, it was used here to assess the completeness and transparency of reporting. One study was appraised as having lower methodological quality [42*], which may limit the strength of its findings; however, its results were consistent with those of the higher-quality studies. Lastly, calculating odds ratios (ORs) from original data can introduce potential for error. We mitigated this by double-checking participant numbers and using MedCalc for statistical calculations. For example, in Mousavi et al. [37*], the authors reported that they had 110 women in the control group where 42 (47.2%) women gave birth vaginally. We assumed the frequency to be correct, and thus reported that in Table 2. However, 42/110 is 38.2%. If 47.2% is correct, then they only had a total of 89 participants in the control group, which would impact the CIs to be insignificant (OR = 1.18, CI = 0.8–1.7.8.7). This discrepancy highlights a broader issue: several included studies, particularly the cross-sectional study, had incomplete reporting of key methodological details, which then limits transparency and interpretability of the findings. Future studies should adhere to the established reporting guidelines to improve quality and comparability.

## Conclusion

In the current review, NS for labour augmentation was safe and effective in a majority of studies. NS reduced synthetic oxytocin usage, increased contraction frequency, shortened the duration of labour stage one, increased Bishop scores, increased the chance of having a spontaneous vaginal birth, and was safe for mother and baby to use with few and reversible side effects. NS may offer a favourable safety profile compared with synthetic oxytocin, though direct comparative evidence is limited and effectiveness appears variable. Clinically, NS presents a promising, low-cost, non-pharmaceutical option that can be initiated by the birthing person, or midwife in various settings, including home births, low-resource environments, and hospitals. Its potential to reduce synthetic oxytocin usage and support physiological labour makes it especially relevant in contexts where medical interventions are either unavailable or undesired. However, given the limited sample sizes and heterogeneity in protocols and measured outcomes, the current evidence is insufficient to support universal routine implementation. Further large-scale randomised controlled trials are needed, preferably using similar control and intervention groups and measuring similar outcomes. Future research should develop protocols for optimal clinical use of NS.

## Supplementary Information

Supplementary Material 1.

## Data Availability

All data generated or analysed during this study are included in this published article [and its supplementary information files].

## Abbreviations

NS: Nipple stimulation
CG: Control group
OI: Oxytocin infusion

## References

[1] Chong YS, Kwek KY. Safer childbirth: avoiding medical interventions for non-medical reasons. Lancet. 2010;375(9713):440–2. 20071023 10.1016/S0140-6736(10)60055-4

[2] Olza I, Leahy-Warren P, Benyamini Y, et al. Women’s psychological experiences of physiological childbirth: a meta-synthesis. BMJ Open. 2018. 10.1136/bmjopen-2017-020347. 30341110 10.1136/bmjopen-2017-020347 PMC6196808

[3] World Health Organization. WHO recommendations: intrapartum care for a positive childbirth experience. 2018. https://www.who.int/publications/i/item/9789241550215. 30070803

[4] Maaløe N, Kujabi ML, Nathan NO, Skovdal M, Dmello BS, Wray S, et al. Inconsistent definitions of labour progress and over-medicalisation cause unnecessary harm during birth. BMJ (Clinical research ed). 2023;383:e076515. 10.1136/bmj-2023-076515. 38084433 10.1136/bmj-2023-076515 PMC10726361

[5] Daly D, Minnie KCS, Blignaut A, Blix E, Vika Nilsen AB, Dencker A, et al. How much synthetic oxytocin is infused during labour? A review and analysis of regimens used in 12 countries. PLoS One. 2020;15(7):e0227941. 10.1371/journal.pone.0227941. 32722667 10.1371/journal.pone.0227941 PMC7386656

[6] Budden. A., Chen. LJY., Henry. A. High‐dose versus low‐dose oxytocin infusion regimens for induction of labour at term. Cochrane Database of Systematic Reviews. 2014;10. 10.1002/14651858.CD009701.pub2. PMID: 25300173. PMCID: PMC8932234 10.1002/14651858.CD009701.pub2 PMC8932234 25300173

[7] Brüggemann, C., Carlhäll, S., Grundström, H., Ramö Isgren, A., & Blomberg, M. Cumulative oxytocin dose in spontaneous labour - Adverse postpartum outcomes, childbirth experience, and breastfeeding. European journal of obstetrics, gynecology, and reproductive biology. 2024;295, 98–103. https://doi-org.proxy.kib.ki.se/ 10.1016/j.ejogrb.2024.01.040. 10.1016/j.ejogrb.2024.01.040 38350309

[8] Grotegut CA, Paglia MJ, Johnson LN, Thames B, James AH. Oxytocin exposure during labour among women with postpartum hemorrhage secondary to uterine atony. American journal of obstetrics and gynecology. 2011;204(1):56-e1. 10.1016/j.ajog.2010.08.023 PMC3018152 21047614

[9] Johnson C. The political “nature” of pregnancy and childbirth. Canadian Journal of Political Science/Revue canadienne de science politique. 2008;41(4):889–913. 10.1017/S0008423908081079.

[10] Laube D. Induction of labour. Clin Obstet Gynecol. 1997;40(3):485–95. 9328728 10.1097/00003081-199709000-00006

[11] Hanley GE, Munro S, Greyson D, et al. Diagnosing onset of labour: a systematic review of definitions in the research literature. BMC Pregnancy Childbirth. 2016;16:71. 10.1186/s12884-016-0857-4. 27039302 10.1186/s12884-016-0857-4 PMC4818892

[12] Kavanagh, J., Kelly, A. J., & Thomas, J. Breast stimulation for cervical ripening and induction of labour. The Cochrane database of systematic reviews. 2005;2005(3), CD003392. https://doi-org.proxy.kib.ki.se/ 10.1002/14651858.CD003392.pub2. 10.1002/14651858.CD003392.pub2 PMC8713553 16034897

[13] McAdow, M. E., Tortal, D., Shabanova, V., & Son, M. Nipple stimulation therapy versus intravenous oxytocin and associated plasma oxytocin concentration during labour induction. American journal of obstetrics & gynecology MFM, 101307. Advance online publication. 2024.https://doi-org.proxy.kib.ki.se/ 10.1016/j.ajogmf.2024.101307. 10.1016/j.ajogmf.2024.101307 38331190

[14] Adams, F. The Genuine Works of Hippocrates, second vol. London: Sydenham Society. American College of Obstetricians and Gynecologists (ACOG). 1849.

[15] Hodgson ZG, Latka P. Canadian registered midwives’ experiences with nipple stimulation: an exploratory survey in British Columbia and Ontario. J Obstet Gynaecol Can. 2020;42(7):861–7. 32430184 10.1016/j.jogc.2019.12.010

[16] Iida M, Katoka Y, Eto H, Tadokoro Y, Masuzawa Y, Yaju Y, et al. The outline of “Japan Academy of Midwifery: Evidence-based guidelines for midwifery care in pregnancy and childbirth–2016 edition.” Journal of Japan Academy of Midwifery. 2018;32(1):73–80.

[17] Buckley SJ. Executive summary of hormonal physiology of childbearing: evidence and implications for women, babies, and maternity care. J Perinat Educ. 2015;24(3):145–53. 26834435 10.1891/1058-1243.24.3.145 PMC4720867

[18] Uvnäs-Moberg K, Ekström-Bergström A, Berg M, et al. Maternal plasma levels of oxytocin during physiological childbirth – a systematic review with implications for uterine contractions and central actions of oxytocin. BMC Pregnancy Childbirth. 2019;19:285. 10.1186/s12884-019-2365-9. 31399062 10.1186/s12884-019-2365-9 PMC6688382

[19] Adewole IF, Franklin O, Matiluko AA. Cervical ripening and induction of labour by breast stimulation. Afr J Med Med Sci. 1993;22(4):81–5. 7839936

[20] Salmon, Y. M., Kee, W. H., Tan, S. L., & Jen, S. Ws. Cervical ripening by breast stimulation. Obstetrics and gynecology. 1986;67(1):21–24. 3940333

[21] Singh N, Tripathi R, Manikya Mala Y, Niharika Y. Breast Stimulation in Low-Risk Primigravidas at Term: Does It Aid in Spontaneous Onset of Labour and Vaginal Delivery? A Pilot Study. BioMed Research International. 2014;2014(695037):6. 10.1155/2014/695037. 10.1155/2014/695037 PMC4265511 25525601

[22] Abedi P, Jahanfar S, Namvar F, Lee J. Breastfeeding or nipple stimulation for reducing postpartum hemorrhage in the third stage of labour. Cochrane Database of Systematic Reviews. 2016(1). 10.1002/14651858.CD010845.pub2Accessed. 10.1002/14651858.CD010845.pub2 PMC6718231 26816300

[23] Almutairi, W.M. Literature Review: Physiological Management for Preventing Postpartum Hemorrhage. Healthcare. Perinatal and Neonatal Medicine. 2021;9(658). 10.3390/healthcare9060658. 10.3390/healthcare9060658 PMC8227540 34073073

[24] Chua S, Arulkumaran S, Lim I, Selamat N, Ratnam SS. Influence of breastfeeding and nipple stimulation on postpartum uterine activity. British Journal of Obstetrics and Gynaecology. 1994;101(9):704–5. 10.1111/j.1471-0528.1994.tb11950.x. PMID: 7947531. 10.1111/j.1471-0528.1994.tb11950.x 7947531

[25] Huddleston JF, Sutliff G, Robinson D. Contraction stress test by intermittent nipple stimulation. Obstet Gynecol. 1984;63(5):669–73 PMID: 6717870. 6717870

[26] Palmer SM, Martin JN, Moreland ML, Ewing J, Bucovaz ET, Morrison JC. Contraction stress test by nipple stimulation: efficacy and safety. South Med J. 1989;79(9):1102–5. 10.1097/00007611-198609000-00015. PMID: 3749994. 10.1097/00007611-198609000-00015 3749994

[27] Popay J, Roberts H, Sowden A, Petticrew M, Arai L, Rodgers M, et al. Guidance on the conduct of narrative synthesis in systematic reviews. ESRC methods programme. 2006;15(1):047–71.

[28] Van Dijk, S. H. B., Brusse-Keizer, M. G. J., Bucsan, C. C., van der Palen, J., Doggen, C., C. J. M., & Lenferink, A. Artificial intelligence in systematic reviews: promising when appropriately used. BMJ open. 2023;13(7), e072254. 10.1136/bmjopen-2023-072254. 10.1136/bmjopen-2023-072254 PMC10335470 37419641

[29] Critical Appraisal Skills Programme. (February 2024). Critical appraisal checklists. https://casp-uk.net/casp-tools-checklists/.

[30] JBI Critical Appraisal Tools. (12 mars 2024). Critical appraisal tools. https://jbi.global/critical-appraisal-tool.

[31] STROBE. (12 mars 2024). STROBE checklists. https://www.strobe-statement.org/checklists/.

[32] Braun V, Clarke V. Using thematic analysis in psychology. Qualitative research in psychology. 2006;3(2):77–101.

[33] MedCalc. MedCalc software. [Statistical mathematical program]. 2024. https://www.medcalc.org/calc/odds_ratio.php.

[34] *Demirel G, Guler H. The Effect of Uterine and Nipple Stimulation on Induction With Oxytocin and the Labour Process. Worldviews Evid Based Nursing. 2015;12(5):273–80. 10.1111/wvn.12116. PMID: 26444882. 10.1111/wvn.12116 26444882

[35] *Frager NB, Miyazaki FS. Intrauterine monitoring of contractions during breast stimulation. Obstetrics and gynecology. 1987;69(5):767–9 PMID: 3574804. 3574804

[36] *Ibrahim h, Ghattas VN, El-Shabory N. Effect of nipple and uterine stimulation on the progress of labour among primiparous women. International Journal of Novel Research in Healthcare and Nursing. 2021;8(2):169–80.

[37] *Mousavi, S., Rouhollahi, B., Zakariya, N. A., Bastani Alamdari, P., & Nikniaz, L. Evaluating the effect of nipple stimulation during labour on labour progression in term pregnant women. Journal of obstetrics and gynaecology : the journal of the Institute of Obstetrics and Gynaecology. 2022;42(5), 994–998. https://doi-org.proxy.kib.ki.se/10.1080/01443615.2021.1980515. 10.1080/01443615.2021.1980515 34927542

[38] *Suja, M. A study to evaluate the effectiveness of nipple stimulation for progress of labour during first stage, among primigravida mothers in a selected hospitals at Tirunelveli, Tamil Nadu. [Master thesis, Medical University of Chennai]. 2015. http://repository-tnmgrmu.ac.in/697/1/3003210sujaj.pdf.

[39] *Curtis, P., Resnick, J. C., Evens, S., & Thompson, C. J. A comparison of breast stimulation and intravenous oxytocin for the augmentation of labour. Birth (Berkeley, Calif.). 1999;26(2):115–122. https://doi-org.proxy.kib.ki.se/10.1046/j.1523-536x.1999.00115.x. 10.1046/j.1523-536x.1999.00115.x 10687576

[40] *Jhirad A, Vago T. Induction of labor by breast stimulation. Obstetrics and gynecology. 1973;41(3):347–50. 4688251

[41] *Stein JL, Bardeguez AD, Verma UL, Tegani N. Nipple stimulation for labour augmentation. J Reprod Med. 1990;35(7):710–4. 2198350

[42] *Young, J. T., & Poppe, C. A. Breast pump stimulation to promote labour. MCN. The American journal of maternal child nursing. 1987;12(2), 124–126. https://doi-org.proxy.kib.ki.se/10.1097/00005721-198703000-00009. 10.1097/00005721-198703000-00009 3104719

[43] *Tal Z, Frankel ZN, Ballas S, Olschwang D. Breast electrostimulation for the induction of labour. Obstet Gynecol. 1988;72(4):671–4. 3262208

[44] Uvnäs-Moberg K, Handlin L. Self-soothing behaviors with particular reference to oxytocin release induced by non-noxious sensory stimulation. Front Psychol. 2015;5:116675. 10.3389/fpsyg.2014.01529 PMC4290532 25628581

[45] Blix E, Kumle M, Kjærgaard H, Øian P, Lindgren HE. Transfer to hospital in planned home births: a systematic review. BMC Pregnancy Childbirth. 2014;14:179. 24886482 10.1186/1471-2393-14-179 PMC4069085

[46] Selin L, Almström E, Wallin G, Berg M. Use and abuse of oxytocin for augmentation of labour. Acta Obstet Gynecol Scand. 2009;88(12):1352–7. 10.3109/00016340903358812. 19878049 10.3109/00016340903358812

[47] Marx Delaney, M., Kumar, V., Katlita, T., Neal, BJKetchum, R., Molina, RL., Singh, S., & Semrau, K. E. Modification of oxytocin use through a coaching-based intervention based on the WHO Safe Childbirth Checklist in Uttar Pradesh, India: a secondary analysis of a cluster randomised controlled trial. BJOG : an international journal of obstetrics and gynaecology. 2021;129(4), 675. https://doi-org.proxy.kib.ki.se/10.1111/1471-0528.17004. 10.1111/1471-0528.16856 34363293

[48] Hoskins NN, Stark EL, Zullo F, Son M, Berghella V. The impact of inpatient nipple stimulation on labor duration: a systematic review and meta-analysis. Am J Obstet Gynecol MFM. 2025;7(4):101650. 10.1016/j.ajogmf.2025.101650. 39978435 10.1016/j.ajogmf.2025.101650

[49] Abu Shqara R, Goldinfeld G, Assulyn T, Sgayer I, Ganem N, Lowenstein L, et al. Breast stimulation vs low dose oxytocin for labor augmentation in women with a previous cesarean delivery, a randomized controlled trial. Am J Obstet Gynecol MFM. 2025;7(5):101658. 10.1016/j.ajogmf.2025.101658. 40054666 10.1016/j.ajogmf.2025.101658

[50] Friedman E. Synthetic oxytocin; critical evaluation in labor and post partum. American journal of obstetrics and gynecology. 1957;74(5):1118–24. 10.1016/0002-9378(57)90170-9. 13469911

[51] Marshall C. The nipple stimulation contraction stress test. J Obstet Gynecol Neonatal Nurs. 1986;15(6):459–62. 10.1111/j.1552-6909.1986.tb01423.x. 3641897 10.1111/j.1552-6909.1986.tb01423.x

[52] Narasimhulu, D. M., & Zhu, L. Uterine Tachysystole with Prolonged Deceleration Following Nipple Stimulation for labour Augmentation. Kathmandu University medical journal (KUMJ). 2015;13(51), 268–270. https://doi-org.proxy.kib.ki.se/10.3126/kumj.v13i3.16820. 10.3126/kumj.v13i3.16820 27180376

[53] Segal, S., Gemer, O., Zohav, E., Siani, M., & Sassoon, E. Evaluation of breast stimulation for induction of labour in women with a prior cesarean section and in grandmultiparas. Acta obstetricia et gynecologica Scandinavica. 1995;74(1), 40–41. https://doi-org.proxy.kib.ki.se/10.3109/00016349509009941. 10.3109/00016349509009941 7856430

[54] Likis, F. E., Andrews, J. C., Collins, M. R., Lewis, R. M., Seroogy, J. J., Starr, S. A., Walden, R. R., & McPheeters, M. L. Nitrous Oxide for the Management of labour Pain. Agency for Healthcare Research and Quality (US). 2012. 23016161

[55] Anderson D. A review of systemic opioids commonly used for labour pain relief. J Midwifery Womens Health. 2011;56(3):222–39. 21535371 10.1111/j.1542-2011.2011.00061.x

[56] Anim-Somuah M, Smyth RM, Cyna AM, Cuthbert A .Epidural versus non-epidural or no analgesia for pain management in labour. Cochrane Database Syst Rev. 2018;21;5(5):CD000331 10.1002/14651858.CD000331.pub4 PMID: 29781504; PMCID: PMC6494646 10.1002/14651858.CD000331.pub4 PMC6494646 29781504

[57] Mahmoodi F, Noroozi M, Mehr LA, Beigi M. Breastfeeding and its outcome in women receiving epidural analgesia for childbirth. Iran J Nurs Midwifery Res. 2019;24(5):355–9. 10.4103/ijnmr.IJNMR_219_18. 31516521 10.4103/ijnmr.IJNMR_219_18 PMC6714128

[58] Katircioglu K, Hasegeli L, Ibrahimhakkioglu HF, Ulusoy B, Damar H. A retrospective review of 34,109 epidural anesthetics for obstetric and gynecologic procedures at a single private hospital in Turkey. Anesth Analg. 2008;107(5):1742–5. 10.1213/ane.0b013e31817bd11f. 18931241 10.1213/ane.0b013e31817bd11f

[59] Vincent RD, Chestnut DH. Epidural analgesia during labour. American family physician. 1998;58(8):1785–92. 9835854

[60] Neimann Rasmussen L, Montgomery P. The prevalence of and factors associated with inclusion of non-English language studies in Campbell systematic reviews: a survey and meta-epidemiological study. Syst Rev. 2018;7:129. 10.1186/s13643-018-0786-6. 30139391 10.1186/s13643-018-0786-6 PMC6107944

[61] Beiranvand SP, Akbari S, Azhari S, Birjandi M. A comparison of the effect of nipple stimulation and oxytocin infusion on the duration of phases of labour. Journal of Kermanshah University of Medical Sciences. 2009;13(2):e79800.

[62] Lestari RH, Aprilia E. Asuhan Kebidanan Pada Ibu Bersalin Dengan Rangsangan Puting Susu Di Bpm Lilik Kustono Diwek Jombang. Strada Jurnal Ilmiah Kesehatan. 2017;6(2):38–42. 10.30994/sjik.v6i2.7.

[63] Mustofa LA, Nuraviah E. Efektifitas Nipple Stimulation Dalam Mencegah Kala II Lama Pada Persalinan Pervaginam. In Prosiding Seminar Penelitian Kesehatan. 2020;2(1):53-8.

[64] Handajani, S. R., & Astuti, K. E. W. Pengaruh Teknik Stimulasi Puting Susu Terhadap Lama Persalinan Kala I. Interest : Jurnal Ilmu Kesehatan. 2016;5(2):193–199. 10.37341/interest.v5.

