# Supplementary material for: Nipple stimulation for labour augmentation: evidence from randomised and quasi-experimental studies
Source: BMC Pregnancy Childbirth. 2025 Dec 1;25:1285. doi: 10.1186/s12884-025-08393-3 (PMC12667179; doi:10.1186/s12884-025-08393-3)
Supplement: Supplementary file 1 — Supplementary Material 1. [file 12884_2025_8393_MOESM1_ESM.docx]

# **SUPPLEMENTARY FILES**

**Supplementary file 1 - Final search**

| Database | Search word | Hits |
| --- | --- | --- |
| **PubMed**  2024.02.15 | (((((((((((breast[MeSH Terms]) OR (nipple[MeSH Terms])) OR (nipple stimulation[Title/Abstract])) OR (breast stimulation[Title/Abstract])) OR (breast pump)[Title/Abstract])) AND (((((((((((((((((((parturition[MeSH Terms])) OR (term birth*[MeSH Terms])) OR (delivery, obstetric[MeSH Terms])) OR (uterine contraction*[MeSH Terms])) OR (labor, second stage[MeSH Terms])) OR (labor, first stage[MeSH Terms])) OR (uterus[MeSH Terms])) OR (cervix[MeSH Terms])) OR (labor[Title/Abstract])) OR (labour[Title/Abstract])) OR (contraction*[Title/Abstract])) OR ("cervical ripening"[Title/Abstract])) OR ("cervical dilation"[Title/Abstract])) OR (childbirth[Title/Abstract])) OR (birth[Title/Abstract])) OR (delivery[Title/Abstract])) OR (parturition[Title/Abstract])))) NOT (((((cancer[Title/Abstract]) OR (tumour[Title/Abstract])) OR (lactation[Title])) OR (breastfeeding[Title])) OR (neonatal[Title]))) NOT (("stress test"[Title]))) NOT (((infant[Title]) OR (newborn[Title])) OR (baby[Title]))) NOT (intercourse[Title]) | 1242 |
| **Cinahl**  2024.02.15 | (MH "Childbirth+") OR (MH "Labor") OR (MH "Cervix Dilatation and Effacement") OR (MH "Uterine Contraction") OR (MH "Labor Stages+") OR (MH "Uterus") OR (MH "Cervix") OR (MH "Delivery, Obstetric") OR (MH "Vaginal Birth+") OR (MH "Intrapartum Care") OR (MH "Management of Labor") OR ( labor OR labour OR contraction* OR "cervical ripening" OR "cervical dilation" OR childbirth* OR birth* OR delivery* OR parturition* ) OR TI ( labor* OR labour* OR contraction* OR "cervical ripening" OR "cervical dilation" OR childbirth* OR birth* OR delivery* OR parturition* )  AND AB ( "nipple stimulation" OR "breast stimulation" OR "breast pump" ) OR TI ( "nipple stimulation" OR "breast stimulation" OR "breast pump" ) OR (MH "Nipples") OR (MH "Breast") | 421 |
| **Web of science**  2024.02.15 | breast stimulation" (Topic) or "nipple stimulation" (Topic) or "breast pump" (Topic) AND labor* (Topic) or labour* (Topic) or contraction* (Topic) or "cervical ripening" (Topic) or "cervical dilation" (Topic) or childbirth* (Topic) or birth* (Topic) or delivery* (Topic) or parturition* (Topic) | 205 |
| **PsychInfo** 2024.02.15 | DE "Birth" OR DE "Pregnancy Outcomes" OR DE "Labor (Childbirth)" OR DE "Natural Childbirth" OR DE "Midwifery" OR ((DE "Labor (Childbirth)" OR DE "Intrapartum Period") AND (DE "Uterus" OR DE "Cervix" OR DE "Placenta" OR DE "Cervix")) OR (DE "Obstetrics") OR ( labor* OR labour* OR contraction* OR "cervical ripening" OR "cervical dilation" OR childbirth* OR birth* OR delivery* OR parturition* ) OR TI ( labor* OR labour* OR contraction* OR "cervical ripening" OR "cervical dilation" OR childbirth* OR birth* OR delivery* OR parturition* )AND DE "Breast" OR "nipple stimulation" OR "breast stimulation" OR "breast pump" | 94 |
| **Google scholar**  2024.02.08 | ("nipple stimulation" OR "breast pump stimulation" OR "breast stimulation") AND (labor* OR labour* OR contraction* OR "cervical ripening*" OR "cervical dilation*" OR childbirth* OR birth* OR delivery* OR parturition*) | 2850 |
| **Endnote**  2024.02.15 |  | **Total hits:** 1962  **Duplicates:**  91st |

**Supplmentary file 2 - test searches**

| Database or AI tool | Search word | Hits |
| --- | --- | --- |
| **consensus.app**  **2024.01.29** | “nipple stimulation for labor augmentation” | 10 |
| **consensus.app**  **2024.01.29** | “breast stimulation for labour augmentation” | 10 |
| **Pubmed**  **2024.02.01** | ("nipple stimulation" OR "breast pump stimulation" OR "breast stimulation" OR "breast pump") AND ("labour progression" OR "labour augmentation" OR "contraction*" OR "childbirth" OR "labour" OR delivery") | 177 |
| **PubMed**  **2024.02.01** | ("nipple stimulation" OR "breast stimulation" OR "breast pump stimulation") AND ("labor" OR "labour" OR "labour augmentation" OR "labor augmentation" OR "oxytocin infusion" OR "labour progression" OR "induction" OR " labour induction" OR "postpartum hemorrhage" OR experiences) | 90 |
| **PubMed**  **2024.02.01** | ("nipple stimulation" OR "Breast stimulation" OR "Breast pump" OR "electric pump" OR "hand pump") AND (intrapartum OR "labor process" OR parturition OR Contractions OR Labor OR Labour OR "Labour progress" OR delivery OR cervical ripening) | 213 |
| **Cihnal**  **2024.01.31** | (nipple stimulation OR breast stimulation OR nipples* OR breast pump) AND (cervical ripening OR labour progress OR labour progression OR contractions in labour)  English filter | 21 |
| **Google scholar**  **2024.02.01** | ("nipple stimulation" OR "breast pump stimulation" OR "breast stimulation") AND (labor* OR labour* OR contraction* OR "cervical ripening*" OR "cervical dilation*" OR childbirth* OR birth* OR delivery* OR parturition*) | 2880 |
| **Web of science**  **2024.02.01** | ("nipple stimulation" OR "breast pump stimulation" OR "breast stimulation") AND (labor* OR labour* OR contraction* OR "cervical ripening*" OR "cervical dilation*" OR childbirth* OR birth* OR delivery* OR parturition*) | 91 |

**Supplementary file 3 - Quality assessment checklists**

| **CASP Criteria** | **Yes^*^** | **No^*^** | **Can´t tell^*^** |
| --- | --- | --- | --- |
| 1. Did the study address a clearly focused research question? | 4 |  |  |
| 2. Was the assignment of participants to interventions ramdomised? | 4 |  |  |
| 3.Were all participants who entered the study accounted for at its conclusion? | 4 |  |  |
| 4.Were the participants “blind” to intervention they were given?  Were the investigators “blind” to the intervention they were giving to the participants?  Were the people assessing/analyzing outcome “blinded”? | 1 | 4  3  4 |  |
| 5.Were the study groups similar at the start of the randomised controlled trail? | 4 |  |  |
| 6.Apart from the experimental intervention, did each study group receive the same level of care? | 4 |  |  |
| 7.Were the effects of intervention reported comprehensively? | 4 |  |  |
| 8.Was the precision of the estimate of the intervention or treatment effect reported? | 4 |  |  |
| 9.Do the benefits of the experimental intervention outweigh the harms and costs? | 4 |  |  |
| 10.Can the results be applied to your local population/in your context? | 4 |  |  |
| 11. Would the experimental intervention provide greater value to the people in your care than any of the existing interventions? | 4 |  |  |

*number of studies

| **JBI Criteria** | Yes | No | Unclear | Not applicable |
| --- | --- | --- | --- | --- |
| 1. Is it clear in the study what is the ‘cause’ and what is the ‘effect’ (i.e. there is no confusion about which variable comes first)? | 4 |  |  |  |
| 2. Were the participants included in any comparisons similar? | 4 |  |  |  |
| 3. Were the participants included in any comparisons receiving similar treatment/care, other than the exposure or intervention of interest? | 4 |  |  |  |
| 4. Was there a control group? | 2 | 2 |  |  |
| 5. Were there multiple measurements of the outcome both pre and post the intervention/exposure? | 4 |  |  |  |
| 6. Was follow up complete and if not, were differences between groups in terms of their follow up adequately described and analyzed? | 3 |  |  | 1 |
| 7. Were the outcomes of participants included in any comparisons measured in the same way? | 2 |  |  | 2 |
| 8. Were outcomes measured in a reliable way? | 4 |  |  |  |
| 9. Was appropriate statistical analysis used? | 4 | 1 |  |  |

|  | **Item No** | **Strobe Checklist Recommendation** | **Y=Yes; N=No** |
| --- | --- | --- | --- |
| **Title and abstract** | 1 | (*a*) Indicate the study’s design with a commonly used term in the title or the abstract | Y |
|  |  | (*b*) Provide in the abstract an informative and balanced summary of what was done and what was found | N |
| **Introduction** | | |  |
| Background/rationale | 2 | Explain the scientific background and rationale for the investigation being reported | Y |
| Objectives | 3 | State specific objectives, including any prespecified hypotheses | N |
| **Methods** | | |  |
| Study design | 4 | Present key elements of study design early in the paper | Y |
| Setting | 5 | Describe the setting, locations, and relevant dates, including periods of recruitment, exposure, follow-up, and data collection | N |
| Participants | 6 | (*a*) *Cohort study*—Give the eligibility criteria, and the sources and methods of selection of participants. Describe methods of follow-up  *Case-control study*—Give the eligibility criteria, and the sources and methods of case ascertainment and control selection. Give the rationale for the choice of cases and controls  *Cross-sectional study*—Give the eligibility criteria, and the sources and methods of selection of participants | Y  -  - |
|  |  | (*b*) *Cohort study*—For matched studies, give matching criteria and number of exposed and unexposed  *Case-control study*—For matched studies, give matching criteria and the number of controls per case | Y |
| Variables | 7 | Clearly define all outcomes, exposures, predictors, potential confounders, and effect modifiers. Give diagnostic criteria, if applicable | Y |
| Data sources/ measurement | 8* | For each variable of interest, give sources of data and details of methods of assessment (measurement). Describe comparability of assessment methods if there is more than one group | N |
| Bias | 9 | Describe any efforts to address potential sources of bias | N |
| Study size | 10 | Explain how the study size was arrived at | Y |
| Quantitative variables | 11 | Explain how quantitative variables were handled in the analyses. If applicable, describe which groupings were chosen and why | N |
| Statistical methods | 12 | (*a*) Describe all statistical methods, including those used to control for confounding | N |
|  |  | (*b*) Describe any methods used to examine subgroups and interactions | Y |
|  |  | (*c*) Explain how missing data were addressed | N |
|  |  | (*d*) *Cohort study*—If applicable, explain how loss to follow-up was addressed  *Case-control study*—If applicable, explain how matching of cases and controls was addressed  *Cross-sectional study*—If applicable, describe analytical methods taking account of sampling strategy | N  -  - |
|  |  | (*e*) Describe any sensitivity analyses | N |

| **Results** | | |  |
| --- | --- | --- | --- |
| Participants | 13* | (a) Report numbers of individuals at each stage of study—eg numbers potentially eligible, examined for eligibility, confirmed eligible, included in the study, completing follow-up, and analysed | Y |
|  |  | (b) Give reasons for non-participation at each stage | N |
|  |  | (c) Consider use of a flow diagram | N |
| Descriptive data | 14* | (a) Give characteristics of study participants (eg demographic, clinical, social) and information on exposures and potential confounders | N |
|  |  | (b) Indicate number of participants with missing data for each variable of interest | N |
|  |  | (c) *Cohort study*—Summarise follow-up time (eg, average and total amount) | Y |
| Outcome data | 15* | *Cohort study*—Report numbers of outcome events or summary measures over time | Y |
|  |  | *Case-control study—*Report numbers in each exposure category, or summary measures of exposure | - |
|  |  | *Cross-sectional study—*Report numbers of outcome events or summary measures | - |
| Main results | 16 | (*a*) Give unadjusted estimates and, if applicable, confounder-adjusted estimates and their precision (eg, 95% confidence interval). Make clear which confounders were adjusted for and why they were included | N |
|  |  | (*b*) Report category boundaries when continuous variables were categorized | N |
|  |  | (*c*) If relevant, consider translating estimates of relative risk into absolute risk for a meaningful time period | N |
| Other analyses | 17 | Report other analyses done—eg analyses of subgroups and interactions, and sensitivity analyses | Y |
| **Discussion** | | |  |
| Key results | 18 | Summarise key results with reference to study objectives | Y |
| Limitations | 19 | Discuss limitations of the study, taking into account sources of potential bias or imprecision. Discuss both direction and magnitude of any potential bias | Y |
| Interpretation | 20 | Give a cautious overall interpretation of results considering objectives, limitations, multiplicity of analyses, results from similar studies, and other relevant evidence | Y |
| Generalisability | 21 | Discuss the generalisability (external validity) of the study results | Y |
| **Other information** | | |  |
| Funding | 22 | Give the source of funding and the role of the funders for the present study and, if applicable, for the original study on which the present article is based | N |

*Give information separately for cases and controls in case-control studies and, if applicable, for exposed and unexposed groups in cohort and cross-sectional studies.

**Supplementary file 4, presenting the formal data of each included article**

| Nr | Reference; Location;Design; | Aim | Sample size= S; intervention size=I; control group size=C; gestational weeks= G | Intervention duration | Intervention type; control group | Obstetric status prior to intervention; | Primiparas=P; multiparas=M | Informed consent (I); ethical approval (E);declares no conflict of interest (NCI) |
| --- | --- | --- | --- | --- | --- | --- | --- | --- |
| 1. | [Mousavi et al, 2022;](https://www.tandfonline.com/doi/full/10.1080/01443615.2021.1980515)  Iran;  RCT | To assess the effect of nipple stimulation during labour on duration of latent and active phases of labour in the term pregnant women. | S=220; I=110; C=110  G=37+ | Intervention stopped when reaching 5 contractions/min or until delivery | Manual | Latent phase, at least 2 contractions/10 min, dilated 2-3 cm or 50% effaced or ruptured membranes; | P=104; M=116 | I =Yes  E = yes NCI = yes |
| 2. | [Stein et al, 1990;](https://pubmed-ncbi-nlm-nih-gov.proxy.kib.ki.se/2198350/)  USA;  RCT | To evaluate the efficacy of nipple stimulation with a breast pump as compared to oxytocin for augmentation of labor. | S=65; I=17; C=48; G=39+ | The intervention was terminated if there was no alteration in the uterine activity within 30 minutes, otherwise continued until delivery. | Breast pump; Oxytocin infusion | All women requiring labor augmentation. Median dilation 5 cm. | Not specified | I= yes; E= no; NCI=no |
| 3. | [Curtis et al, 1999](https://onlinelibrary.wiley.com/doi/10.1046/j.1523-536x.1999.00115.x);  USA; RCT | To compare the outcomes of breast stimulation compared with oxytocin in augmenting labor in women with ruptured membranes and inadequate labor. | S=79; I=49; C=30;G ≥ 33 | Nipple stimulation discontinued after 60 min if no observed effects, otherwise continued until delivery. | Breast pump; Oxytocin infusion | Ruptured membranes 1-24 hrs, with a maximum of 2 contractions/10 min. | P=48; M=30 | I=yes; E=no; NCI=no |
| 4. | Young & Poppe, 1987; USA; retrospective | To gain more information about BPS (breast pump stimulation), we reviewed the charts of 26 mothers who had used the procedure at our institution. | S=26, G=37-41 | Continued for as long as the woman chose. | Breast pump. | Ruptured membranes, with or without contractions. | P=9; M=17 | I=no; E=no; NCI=no |
| 5. | [Ibrahim et al, 2021;](https://www.noveltyjournals.com/upload/paper/Effect%20of%20Nipple%20and%20Uterine%20Stimulation.pdf) Egypt,  Quasi-experimental | Determine the effect of nipple and uterine stimulation on the progress of labor among primiparous Women. | S=150; I=50; C=50; U= 50; G37+ | Continued until delivery. | Manual; uterine stimulation; control group. | Median Bishop score 8, active phase of labor. | P=150; M=0 | I=yes; E= no; NCI=no |
| 6. | [Suja, 2015](http://repository-tnmgrmu.ac.in/697/1/3003210sujaj.pdf);  India;  Quasi-experiemental | To evaluate the effectiveness of nipple stimulation for progress of labour during first stage, among primigravida mothers in selected hospital at tirunelveli. | S=60; I=30; C=30; G=37-40 | 10 min stimulation with a 10 min break continuously for 2 hours. | Manual | Spontaneous onset of labor; 2 cm dilation; mild contractions. | P=60; M=0 | I=yes; E=no; NCI=no |
| 7. | [Frager & Miyazaki, 1987;](https://pubmed-ncbi-nlm-nih-gov.proxy.kib.ki.se/3574804/) USA; experimenal | To measure accurately with the intrauterine pressure catheter the intensity and frequency of uterine contractions produced by breast stimulation. | S=36; I=0; G=35+ | Stimulation during one hour. | Manual stimulation | Ruptured membranes; 1-4 cm dilation. | Not specified | I=no; E=no; NCI=no |
| 8. | [Tal et al](https://pubmed-ncbi-nlm-nih-gov.proxy.kib.ki.se/3262208/), 1988; Israel; experiemental | We studied breast electrostimulation as a controllable method of initiating labor in 21 women. | S=21; C=0; G=36-42. | At least until the end of labor first stage. | Breast pump | 9 ruptured membranes, 9 requiring induction for medical reasons. Varying Bishop score. | Not specified. | I=no; E=no; NCI= no |
| 9. | [Jhirad & Vago, 1973;](https://pubmed-ncbi-nlm-nih-gov.proxy.kib.ki.se/4409835/) Israel; experimental |  | S=204; C=0; G=Not specified. | Until delivery or until the need for a cesarean section. | Electric breast pump. | 59 with ruptured membranes, 12 with uterine inertia. | P=32; M=28 | I=no; E=no; NCI= no |
| 10. | [Demirel & Guler; 2015;](https://pubmed.ncbi.nlm.nih.gov/26444882/) Turkey;  RCT | Determining the effect of uterine and nipple stimulation on induction with oxytocin and the birth process. | S=390; I=130; U=130; C=130;  G=Not specified | Terminated if no cervical dilation after 2 hours, otherwise continued for at least 8 hours. | Manual stimulation | Inclusion criteria bishop score ≥6, all participants had a bishop score of 8,9-9. | P=195; M=165 | I=yes; E=yes; NCI=no |
